# Exercise-related changes in the anabolic index (testosterone to cortisol ratio) and serum amyloid A concentration in endurance and racehorses at different fitness levels

**DOI:** 10.3389/fvets.2023.1148990

**Published:** 2023-04-17

**Authors:** Jowita Grzędzicka, Izabela Dąbrowska, Katarzyna Malin, Olga Witkowska-Piłaszewicz

**Affiliations:** Department of Large Animal Diseases and Clinic, Institute of Veterinary Medicine, Warsaw University of Life Sciences, Warsaw, Poland

**Keywords:** equine, testosterone, cortisol, T/C index (ratio), biomarkers, sport medicine, overtraining, injuries

## Abstract

Increased training loads in both human and equine athletes are generally implemented to improve their physical performance. These loads are tolerated only within appropriate training periodization with regard to recovery time. Otherwise, training overload causes failure in the systemic adaptation, which at first leads to overreaching, and progressively to overtraining syndrome (OTS). Exercise endocrinology, and anabolic/catabolic balance as an indicator of athlete performance status and OTS has continued to attract attention. In human medicine, changes in testosterone and cortisol levels, as well as the testosterone to cortisol ratio (T/C; anabolic index), are suggested to be sensitive stress markers. However, there is a lack of research investigating these parameters for use in equine sports medicine. The aim of the study was to investigate the differences in testosterone, cortisol, and T/C in response to a single training session in two types of equine sports: endurance and race, together with serum amyloid A (SAA), the main acute phase response indicator of physical effort, and the overall health status in horses. Two groups of horses were enrolled in the study: endurance (*n* = 12) and racehorses (*n* = 32) of different fitness level. Blood samples were obtained before and after the exercise. On average, T increased 2.5 times after the race training in experienced racehorses and dropped in endurance horses regardless the fitness level (*p* < 0.05). In endurance horses, a decrease in T/C occurred after training in inexperienced horses (*p* < 0.05). In racehorses, a T/C decrease occurred in the inexperienced group (*p* < 0.05) and an increase in the experienced (*p* < 0.01). In conclusion, T/C ratio was found to be a potentially reliable indicator of fitness status especially in racing horses. These findings provide insight into the physiological response of the horses to different types of exercise and the potential use of hormone levels as markers of performance and adaptation.

## 1. Introduction

Physical exercise is an effective stress stimulus that induces a physiological reaction toward restoring homeostasis. The stress response is activated when the threshold of this equilibrium is reached, which can be constructive or destructive to an organism (1). This signaling primarily affects the neuroendocrine system; especially the hypothalamic–pituitary–adrenal axis (HPA) which increases secretion of cortisol (C), when stimulated (2). Cortisol is a critical mediator of gluconeogenesis and the catabolic state, necessary to cope with the increased energy demand during exercise targeting many tissues of the body, especially the skeletal muscles (3). Both human and animal research provides evidence that depleted ability of cortisol production results in decreased physical performance, most likely due to hypoglycemia, reduction in glycogen concentration, and low availability of free amino acids pool, whichserves as an additional substrate for oxidation (4–6). Some level of stress is needed for everyday response, and the optimum is called “Eustress” (7). Additionally, the increased catabolism during exercise requires another adaptation to counteract the excessive proteolysis and spare the demanding skeletal muscle. Androgens are responsible for the anabolic progress. It was suggested that testosterone (T) is the factor that primarily promotes the incorporation of amino acids into muscle proteins. The molecular basis of the anti-catabolic action of testosterone may lie in both: (1) the competitive action of the androgen receptor-testosterone complex and glucocorticoid receptor-cortisol complex on the regulatory site of DNA and (2) the reduction of androgen and glucocorticoid receptor expression. Along with the anti-catabolic function, testosterone demonstrates its active anabolic role in multiple ways. Testosterone-induced myogenesis and muscle hypertrophy are promoted by genomic and non-genomic activation of molecular pathways that stimulate muscle’s satellite cells and regulate androgen receptors (8).

Given the interplay of C and T in maintaining anabolic-catabolic balance and their highly sensitive response to acute and chronic exercise, testosterone-to-cortisol ratio (T/C) is a helpful parameter for indication of exercise intensity loaded on the individual and the consequences of this stress in the context of behavioral changes, overreaching, overtraining, strength gain, recovery, and adaptation to the following effort (9–11). The T/C, an absolute number also referred to as the anabolic index, has been shown to drop by more than 30% in human athletes with symptoms of overtraining and poor performance. However, this index cannot be used alone as a threshold for a diagnosis of overtraining syndrome (OTS) (10, 12). Nevertheless, measured in the follow-up manner throughout the training season, it is a valuable biological marker of appropriate progress of athlete performance and accuracy of the implemented training program (12, 13). However, in equine athletes, there is a lack of studies concerning the changes and usefulness of the T/C ratio. There is only one preliminary study confirming that the anabolic index does not change in leisure horses after training season (11).

Together, the hormonal response, and the mild inflammatory process have a highly beneficial effect on the regeneration of tissues damaged by exercise. In addition, the inflammatory process in the muscle is essential for adaption to an increased workload during physical training (14). Induction of stress response that highly disturbs homeostasis may influence acute phase reaction (APR), which is an immediate systemic reaction to the inflammatory process (15). APR is also an indicator of clinical pathologies connected with inflammatory state (16). Acute phase proteins (APPs) constitute valuable biomarkers of the APR, among others C-reactive protein (CRP), serum amyloid A (SAA), haptoglobin, fibrinogen, transferrin, and ceruloplasmin (17). In horses, the most sensitive one is SAA, which is considered the main APP in this species, and whose concentration may increase a thousandfold in APR immediately after stimuli (16). According to the overload principle, repeated exercise that moderately surpasses cellular homeostasis capacity and induces sustainable APR leads to positive remodeling of skeletal muscle tissue and an anti-inflammatory state following appropriate recovery. When resting time is not respected or the training load is too intense, regulation of the pro-inflammatory process might be unbalanced, which consequently leads to OTS (18). Thus, evaluating the SAA changes together with T/C will give additional information about training status.

In equine sports research, methods for health preservation during the training season remain a challenge. Individual adaptation to exercise load is essential for to avoid OTS and consequently, an increase in performance and lower susceptibility to injuries. Since OTS has a progressive appearance in the horse, it can be difficult to diagnose. The early symptoms of OTS include: increased heart rate, weight loss, drop of plasma C after exercise, and increased plasma concentration of gamma-glutamyl transferase (GGT), however, there is not an ideal biomarker. Behavioral score constitutes an auxiliary indicator, however it depends on the behaviorist’s experience and may relate to horses’ personalities (19). Novel, non-invasive techniques are evaluated to obtain the stress level in the horse like infrared thermography (IRT), however, they are still not validated (20, 21).

Early detection of horse maladaptation to training load is essential for OTS prevention and shorter convalescence period (22). Thus, our study is an attempt to evaluate the changes in the anabolic index (T/C ratio) and SAA plasma concentration in two modes of exercise: long-lasting, moderate intensity (endurance) and short, high intensity (race) in horses at different fitness level.

## 2. Materials and methods

### 2.1. Animals

Two groups of horses were enrolled in the study: endurance (*n* = 12) and racehorses (*n* = 32). The inclusion criterion of good health was based on veterinary examination, which included heart rate, mucous membranes (color and moisture), dehydration (measured as the time it takes for a pinched skin fold over the point of the shoulder to flatten), gut sounds, muscle condition and regularity of gait (evaluated in trot). Additionally, basal morphological and biochemical blood examination before and after exercise was performed and no abnormalities were observed.

#### 2.1.1. Endurance horses

Private-owned healthy endurance Arabians (*n* = 12) aged 9–12 years were enrolled in the study. All of them participated in the endurance competition and were divided into two groups: experienced horses which competed at the 120 km distance (*n* = 5; 2 mares and 3 geldings); and inexperienced horses which competed at the 100 km distance (*n* = 7; 4 mares, 3 geldings). They were fed a standard diet designed for endurance horses, which included hay (7.5 kg/horse), oats, oils, and particular concentrate for endurance horses (2–4 kg/horse). The concentrated feed and roughage were given three times per day. They were trained during daily sessions with the exercise load depending on the horse’s condition and increased in time; the sessions with high exercise load were performed every 14–20 days. The speed was 15.4–18.7 km/h in 120 km competition and 13.4–17,3 km/h in 100 km competition. One horse did not finish the 120 km competition in Southern Poland.

#### 2.1.2. Racehorses

The 32 horses were divided into two groups: (1) (*n* = 16) experienced horses after 2–5 training seasons (4–8 years old) – 9 Thoroughbreds (7 mares, 1 stallion and 1 gelding) and 7 Arabians (3 mares and 4 stallions); and (2) (*n* = 16) inexperienced horses (2–4 years old) at the beginning of their first race training season – this group consisted of 9 Thoroughbreds (5 mares, 4 stallions) and 7 Arabians (4 mares and 3 stallions). All horses were kept in the same environmental conditions in central Poland, and trained by one trainer. They were fed with the standard diet designed for racehorses (oats 5.5 kg/horse, meadow hay 7.5 kg/horse, and special concentrate for performance horses). The concentrated feed and roughage were given three times per day.

The horses exercised on the sand, at a speed of about 800 m/min for 800 m. For the untrained horses, it was the first training session with a gallop. The training involved daily sessions of galloping on the sand for 800–1,000 m. High speed exercise sessions took place every 2 days in cycles of 5 days a week.

### 2.2. Blood samples

Peripheral blood was gathered from the jugular vein into sterile K2-ethylenediaminetetraacetic acid (K2-EDTA) tubes for hematological tests and sterile dry tubes for serum analyses using the BD Vaccutainer system (BD, USA). Tubes were centrifuged (3,000× *g*, 15 min), and serum was isolated and stored at −80°C for further analyses.

Blood samples were collected before starting the exercise (1 h before feeding) and immediately after the end of the race training/max. 30 min after the end of the endurance effort, as a part of standard veterinary diagnostic procedures, which is in line with other studies (11, 20, 23, 24). Thus, no approval of the Local Commission for Ethics in Animal Experiments was required, according to the Polish legal regulations and the European directive EU/2010/6.

### 2.3. ELISA

To determine cortisol and testosterone concentration, the available immunoenzymatic commercial assay dedicated and validated for equine species was used (cortisol cat. no.: ELK8554; testosterone cat. no.: ELK8556; ELISA, ELK Biotechnology Co., Ltd., Wuhan, China). Intra-assay precision (precision within an assay): CV% < 8%; inter-assay precision (precision between assays): CV% < 10%.

SAA concentrations were measured using a multispecies but validated for equine species immunoenzymatic commercial assay (cat. no. TP-802; PHASE SAA Assay, Tridelta Ltd., Maynooth, Ireland). Sample dilution was 1:1000 instead of 1:2000 recommended by the manufacturer’s protocol, and the results were appropriately recalculated. The assay, including the dilution, was previously validated for the determination of SAA concentrations in horses (16). The used enzyme immunoassay has an intra-assay precision CV% < 5% and an inter-assay precision CV < 11. The absorbance was measured by Multiscan Reader (Labsystem, Helsinki, Finland) using a Genesis V 3.00 software program.

### 2.4. Statistical analyses

For statistical analysis, the OriginPro 2022 statistics package (OriginLab Corporation, Northampton, MA, USA) was used. The data distribution was assessed by the Shapiro–Wilk test. Comparison of data measured before and after training within the groups was analyzed using paired sample *t*-test or Wilcoxon signed-rank test, depending on the data distribution normality. Statistical significance of parameters’ values differences between groups (experienced and inexperienced horses) was confirmed based on the T-test or Mann-Withney test, depending on the data distribution normality. A value of *p* < 0.05 was considered a threshold for differences’ significance. The availability of data allowed us to investigate the interracial differences for every measured parameter. Dunn’s post-hoc analysis with ANOVA was performed for that purpose.

## 3. Results

### 3.1. Comparison of changes in testosterone levels before and after exercise

#### 3.1.1. Racehorses

**Figure 1:**
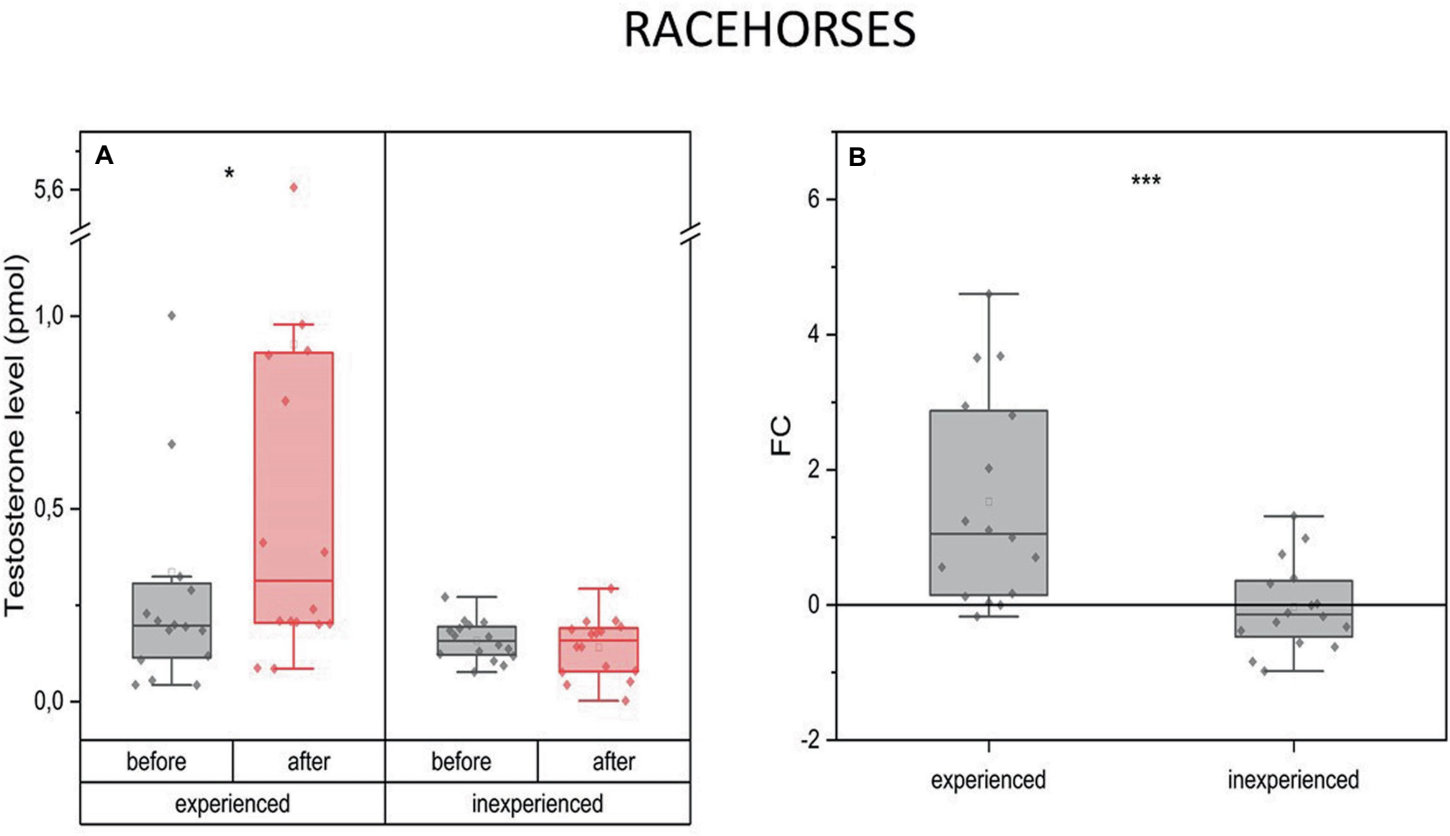
Changes in testosterone plasma level upon training in experienced (*n* = 16) and inexperienced (*n* = 16) racehorses. Box plots (IQR, mean and median; whisker range within 1,5IQR) expressed as differences in molar plasma concentration **(A)** and fold change (FC) **(B)**. Statistically significant differences are indicated by asterisks at *p* < 0.05 (*), *p* < 0.01 (**), and *p* < 0.001 (***).

On average, T levels increased 2.5 times after the race training (mean FC = 15, IQR = 2.7) in the experienced horses, while in the inexperienced horses, they remained on the same level as before the training (mean FC = 002, IQR = 0.8) (Figure 1).

The availability of data allowed us to investigate the interracial differences for every measured parameter. Inexperienced Arabian racehorses show a significant drop of T in response to training (mean FC = −0.5) in comparison to 2-fold increase in experienced Arabians. This was not the case for the Thoroughbred racehorses.

#### 3.1.2. Endurance horses

**Figure 2:**
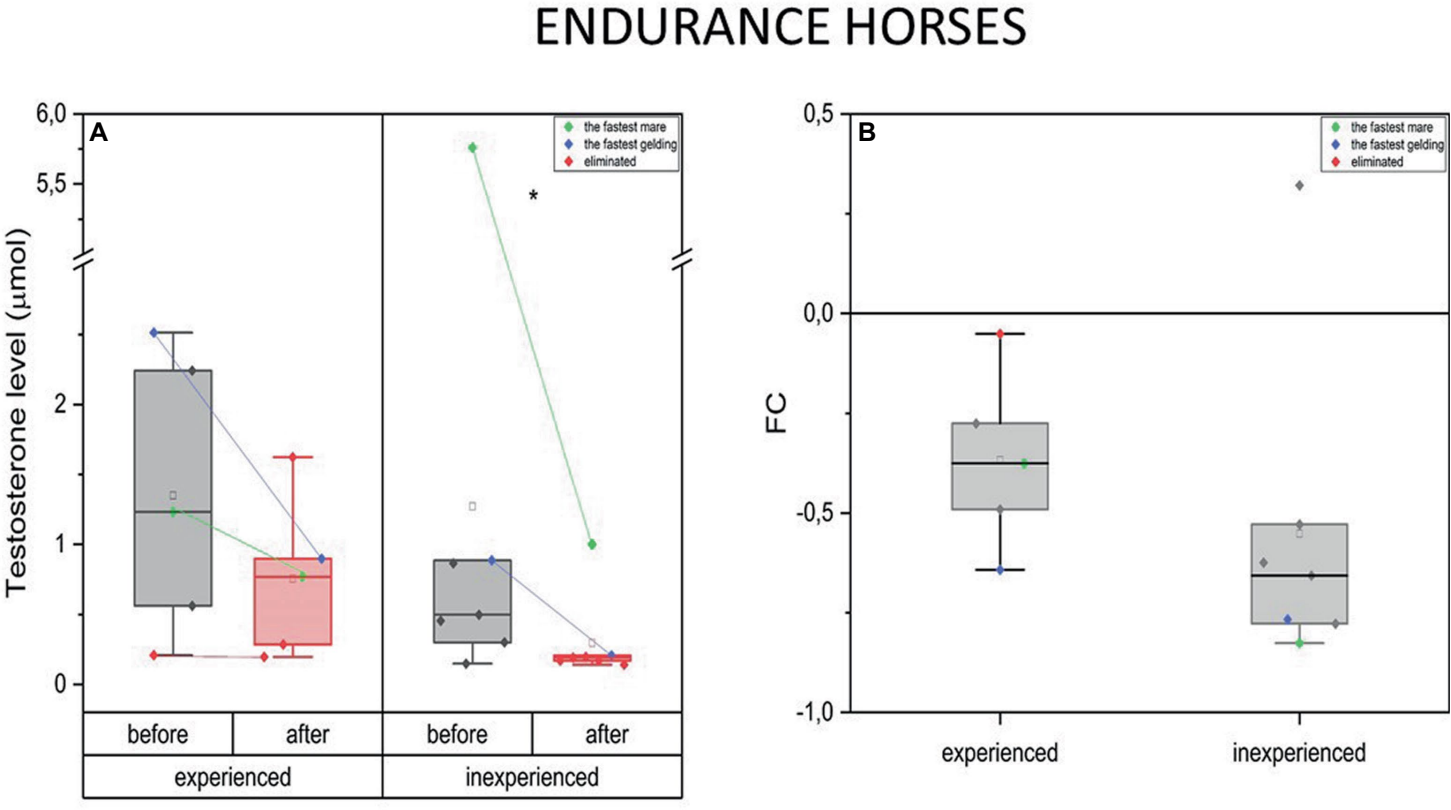
Changes in testosterone plasma level upon training in experienced (*n* = 5) and inexperienced (*n* = 7) endurance horses. Colored data points indicate the fastest mare (green), the fastest gelding (blue), and the eliminated gelding (red). Box plots (IQR, mean and median; whisker range within 1,5 IQR) expressed as differences in molar plasma concentration **(A)** and fold change (FC) **(B)**. Statistically significant differences are indicated by asterisks at *p* < 0.05 (*).

The endurance mode of exercise induced drop in T plasma concentration in the experienced and inexperienced horses (mean FC = −0.37, IQR = 0.2 and –0.55, IQR = 0.2 respectively). The decrease in T concentration after training occurred only in endurance horses after 100 km competition (inexperienced) [*p* < 0.05 (*)] (Figure 2).

The measurements taken before and after training resulted in a decrease in T concentration only in the inexperienced group. The differences in this parameter’s FC between the two groups were not significant (Figure 2B).

Measurements taken in the fastest endurance geldings and mares showed a high basal level of blood testosterone in both experienced and inexperienced endurance groups. Furthermore, the fastest horses experienced a substantial drop in T concentration in response to extraordinary physical effort. This change is greater in the inexperienced group. It should be noted that the eliminated gelding did not exhibit an increase in T levels in its blood either before or after the race (Figure 2B).

### 3.2. Comparison of changes in cortisol levels before and after exercise

#### 3.2.1. Racehorses

**Figure 3:**
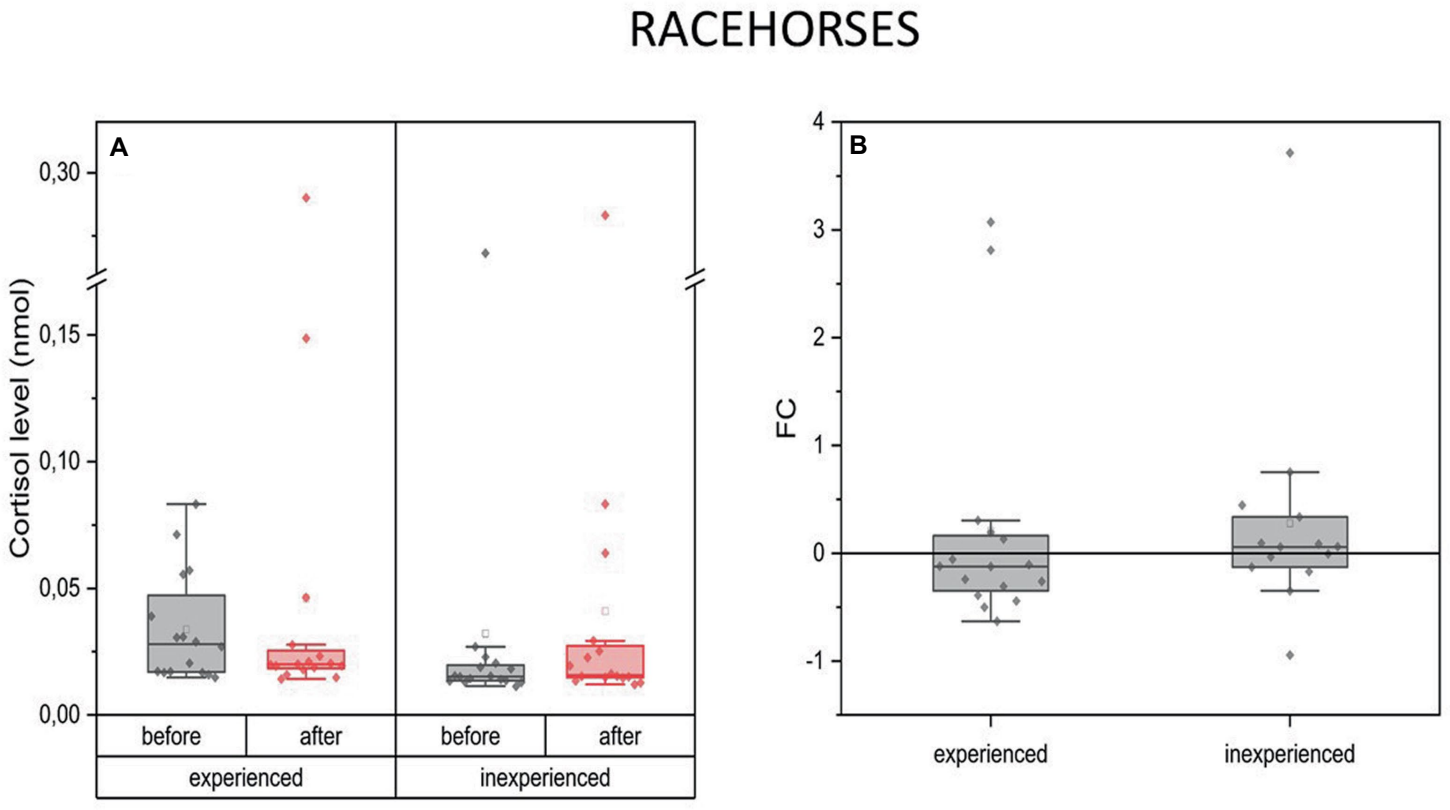
Changes in cortisol plasma concentration upon training in experienced (*n* = 16) and inexperienced (*n* = 16) racehorses. Box plots (IQR, mean and median; whisker range within 1.5IQR) expressed as differences in molar plasma concentration **(A)** and fold change (FC) **(B)**.

Changes in blood C concentration in racing horses were comparable in both experienced and inexperienced horses. There was no difference between the two groups (Figure 3).

#### 3.2.2. Endurance horses

**Figure 4:**
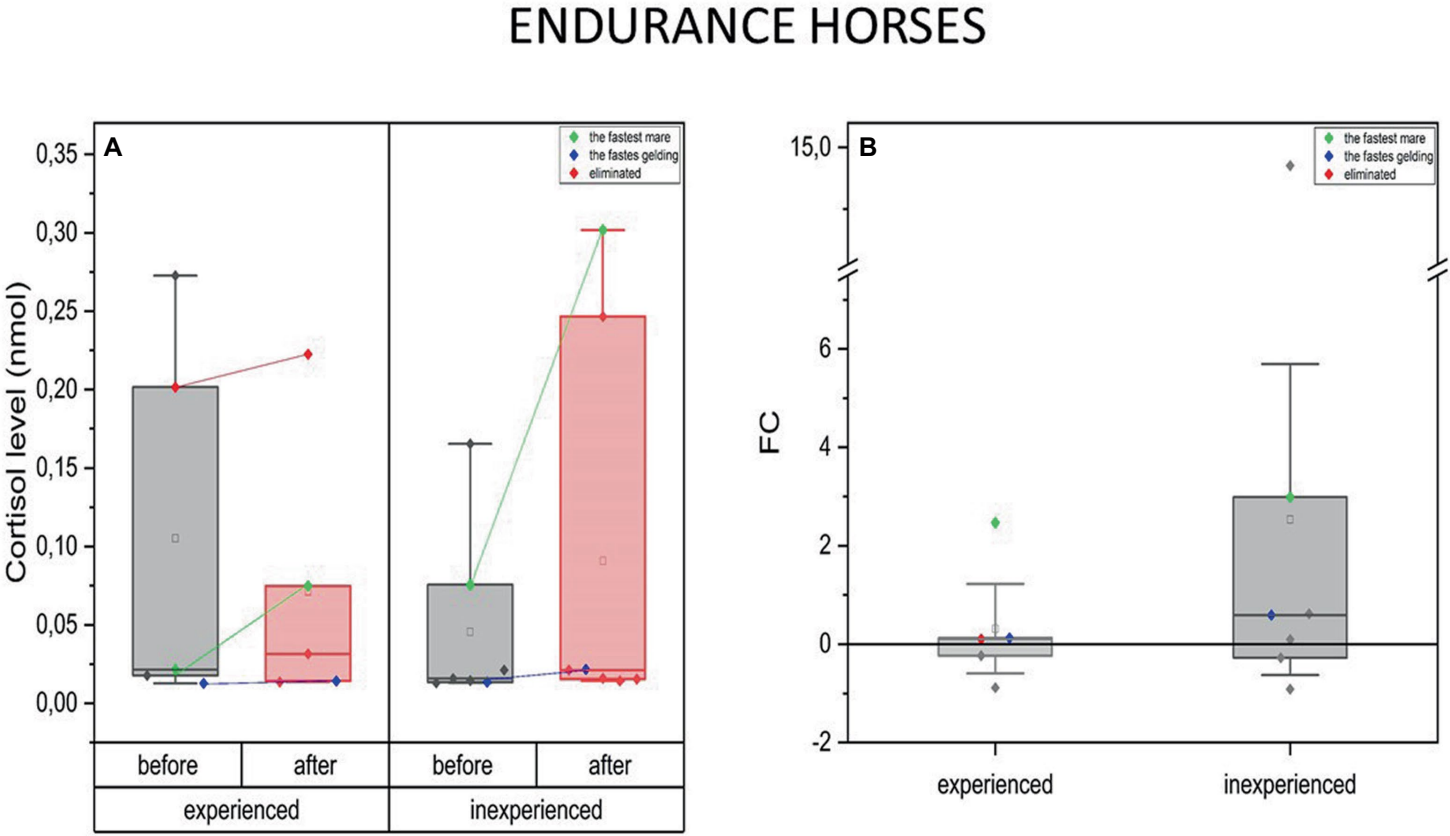
Changes in cortisol plasma concentration upon training in experienced (*n* = 5) and inexperienced (*n* = 7) endurance horses. Colored data points indicate the fastest mare (green), the fastest gelding (blue), and the eliminated gelding (red). Box plots (IQR, mean and median; whisker range within 1.5 IQR) are expressed as differences in molar plasma concentration **(A)** and fold change (FC) **(B)**.

Examining both groups of endurance horses did not reveal changes in blood C concentration in response to exercise. However, the data allowed us to notice interesting relationships (Figure 4).

The difference in C levels for the fastest mare and gelding in the group should be highlighted. At both fitness levels, it can be noticed that high-intensity effort induced none or only a slight change in blood C concentration in male horses. In this case, for the inexperienced gelding, an increase of C by over a half of the previous value (FC = 0.6) was observed (Figure 4B).

Furthermore, C concentration in both the experienced and inexperienced group’s fastest mares increased significantly upon the training’s completion. Similarly to the geldings, the inexperienced fastest mare’s C level increase was greater (FC = 3) compared to the experienced one (FC = 2.5) (Figure 4B).

Data from the eliminated horse provided additional scope. In this case, the C concentration was much higher at the beginning of the measurements, compared to the group’s mean, prior to the race, and remained at the same level in the post-training measurement.

When comparing the experienced and inexperienced horses regarding the degree of changes in blood C levels, the distribution of data within the group of inexperienced animals was more spread out and directed toward an increase in C concentration after the exercise. However, a comparison of exercise-induced C concentration changes between experienced and inexperienced endurance horses did not reveal any significant differences.

### 3.3. Comparison of changes in the anabolic index (T/C ratio) before and after exercise

#### 3.3.1. Racehorses

**Figure 5:**
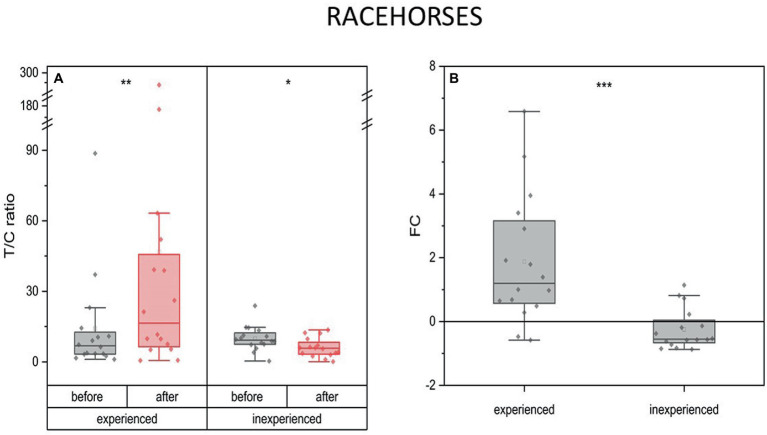
Changes in the anabolic index (T/C ratio) upon training in experienced (*n* = 16) and inexperienced (*n* = 16) racehorses. Box plots (IQR, mean and median; whisker range within 1,5IQR) are expressed as the ratio of hormones’ molar plasma concentration **(A)** and fold change (FC) **(B)**. Statistically significant differences are indicated by asterisks at *p* < 0.05 (*), *p* < 0.01 (**), and *p* < 0.001 (***).

The experienced and inexperienced racehorses presented inverse patterns of change in the T/C ratio in response to training. A decrease was observed in the inexperienced group (mean FC = -0.25, IQR = 0.7) (*p* < 0.05), while in the experienced animals T/C ratio was three times as high as the pre-training ratio (mean FC = 1.88, IQR = 2.6) (*p* < 0.01) (Figure 5).

#### 3.3.2. Endurance horses

**Figure 6:**
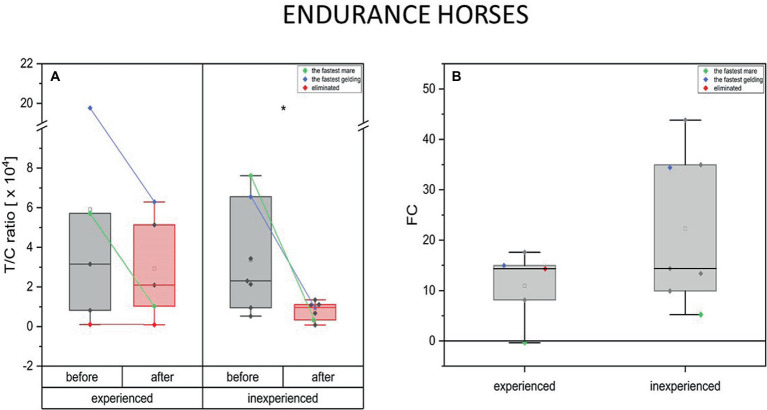
Changes in the anabolic index (T/C ratio) upon training in experienced (*n* = 5) and inexperienced (*n* = 7) endurance horses. Colored data points indicate the fastest mare (green), the fastest gelding (blue), and the eliminated gelding (red). Box plots (IQR, mean and median; whisker range within 1,5IQR) are expressed as differences in molar plasma concentration **(A)** and fold change (FC) **(B)**. Statistically significant differences are indicated by asterisks at *p* < 0.05 (*), *p* < 0.01 (**), and *p* < 0.001 (***).

A decrease in the anabolic index after training was present in both groups of different training advancement in the endurance horses. However, this decrease was significant only in the case of inexperienced horses. In both groups, the fastest mare had a greater decline in the anabolic index compared to the fastest gelding. Moreover, the outstanding athletes experienced a more pronounced drop in the T/C ratio (below the group’s average). Furthermore, the horse that was eliminated demonstrated an unchanged anabolic index (Figure 6).

### 3.4. Comparison of changes in SAA levels before and after exercise

#### 3.4.1. Racehorses

**Figure 7:**
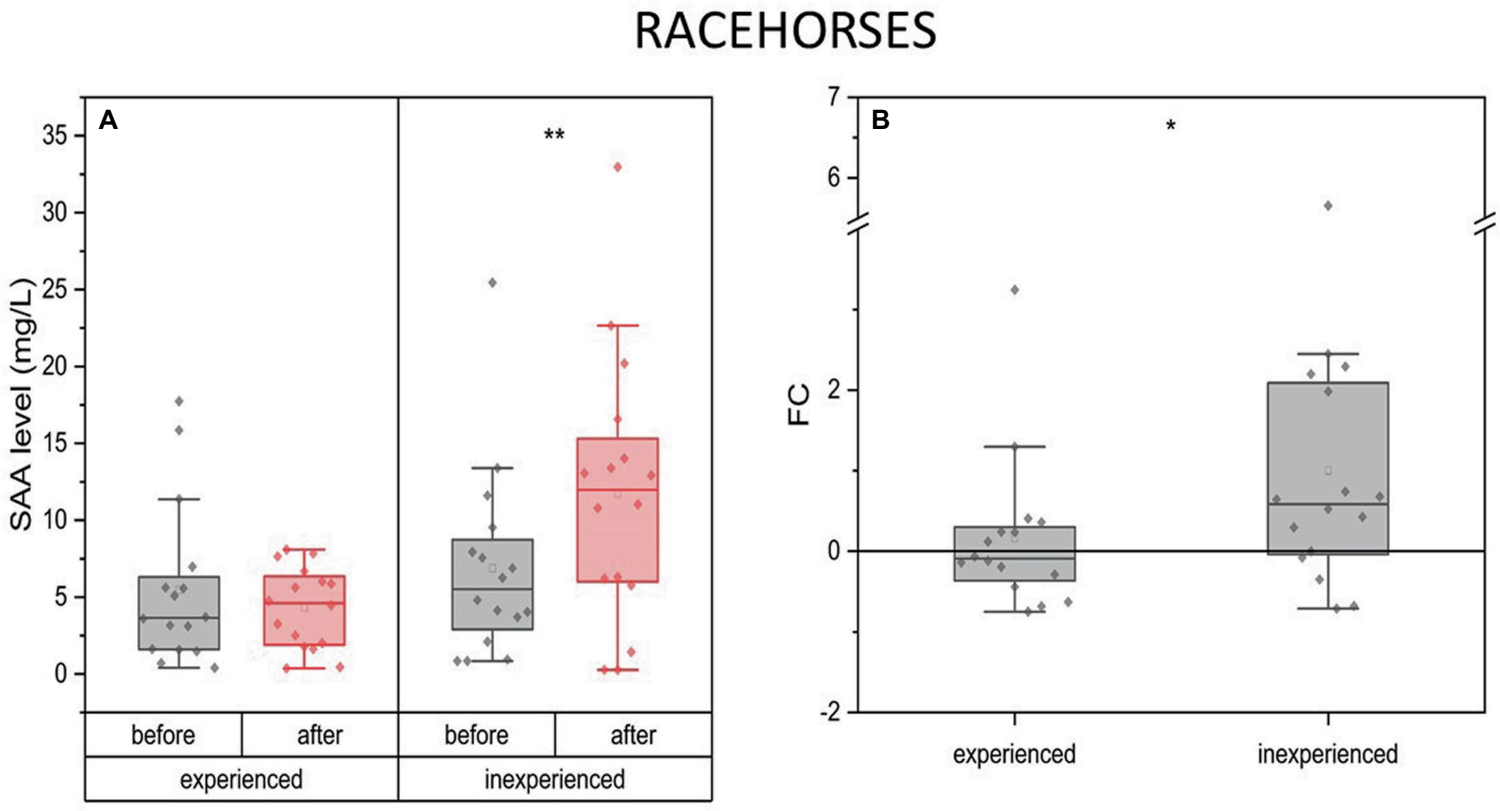
Changes in SAA plasma concentration upon training in experienced (*n* = 16) and inexperienced (*n* = 16) racehorses. Box plots (IQR, mean and median; whisker range within 1,5IQR) are expressed as the ratio of hormones’ molar plasma concentration **(A)** and fold change (FC) **(B)**. Statistically significant differences are indicated by asterisks at *p* < 0.05 (*), *p* < 0.01 (**), and *p* < 0.001 (***).

The SAA concentration did not change after training in the experienced racehorses. However, a significant increase was noted in the inexperienced group (mean FC = 0.58, IQR = 0.8). The degree to which SAA concentration changed during the experiment between these two groups expressed as a fold change also indicated a significant difference (Figure 7).

#### 3.4.2. Endurance horses

**Figure 8:**
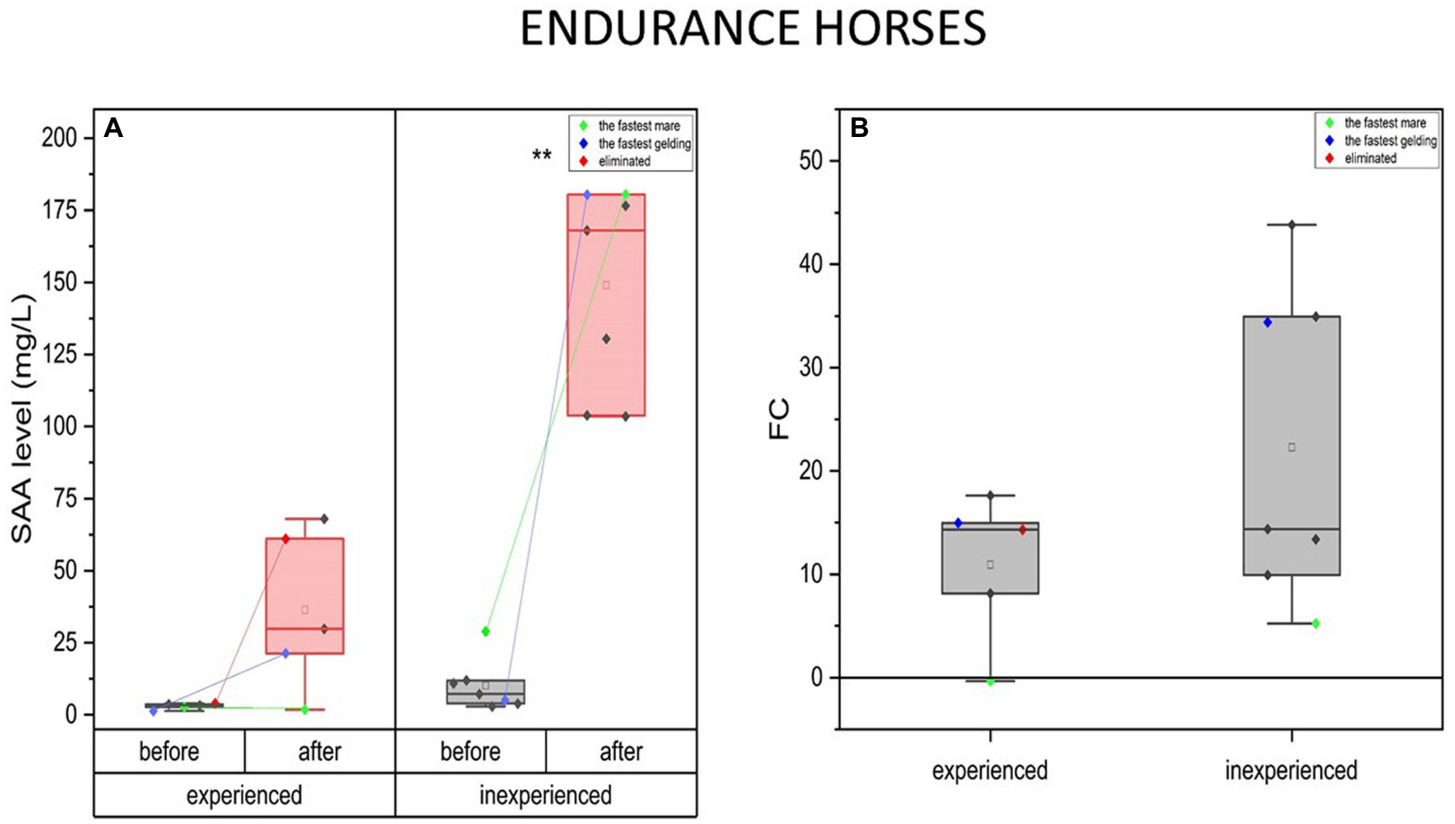
Changes in SAA plasma concentration upon training in experienced (*n* = 5) and inexperienced (*n* = 7) endurance horses. Colored data points indicate the fastest mare (green), the fastest gelding (blue), and the eliminated gelding (red). Box plots (IQR, mean and median; whisker range within 15IQR) are expressed as differences in molar plasma concentration **(A)** and fold change (FC) **(B)**. Statistically significant differences are indicated by asterisks at *p* < 0.05 (*), *p* < 0.01 (**), and *p* < 0.001 (***).

SAA concentrations rose over 10-fold after the endurance training, the same in the experienced (mean FC = 11, IQR = 68) and inexperienced group (FC = 22, IQR = 25). SAA concentration was significantly higher in the inexperienced group, both before (mean = 10 mg/L, IQR = 8) and after the completion (mean = 149 mg/L; IQR = 765) compared to the experienced group (before, mean = 3; IQR = 0.96; after, mean = 36; IQR = 40) (Figure 8).

In the fastest horses, SAA change tendencies were similar in both groups. SAA concentration increase for the inexperienced fastest mare was minor compared to the group average, while the experienced one’s dropped (FC = −0.3). On the contrary, in response to training, the fastest geldings presented much greater increase of SAA blood concentration in the group. This was also the case for the eliminated gelding, for which this parameter’s change was comparable to the fastest gelding in the group. Nevertheless, the degree to which the SAA level changed cannot be considered significant between experienced and inexperienced horses’ groups.

Furthermore, the eliminated horse had a higher basal level of SAA (4 mg/ml) than the average in the experienced group (mean = 3 mg/l; IQR = 0.96 mg/l), but still significantly lower than in the inexperienced horses (mean = 10 mg/l; IQR = 8 mg/l). When comparing the experienced to the inexperienced horses, the FC of SAA presents similar tendencies in the fastest mares and geldings. The increase in SAA was insignificant in the fastest, experienced mare (FC = −0.34) and the lowest in the inexperienced fastest mare (FC = 5.2) compared to the group. The fastest geldings, on the contrary, have one of the highest SAA concentration FC in both groups.

## 4. Discussion

### 4.1. Testosterone

#### 4.1.1. Breed differences

The availability of data allowed us to investigate the interracial differences for every measured parameter (data not shown). It appears that Thoroughbred and Arabians might represent different adaptation mechanisms by means of T secretion in response to exercise. In inexperienced Arabian racehorses, a drop of T in response to training in comparison to 2-fold increase in experienced Arabians occurred. This was not the case for the Thoroughbred racehorses. In this breed, an increase in T level after the race in both groups of athletic advancement was not significant. This might indicate that Thoroughbreds might adapt to race training better, which is reflected in higher anabolic response in the early rest period after the physical effort, even in horses that ran their first season. The above-described differences might find its explanation in physiological differences between Arabians and Thoroughbreds that predispose to types of activity. Compared to the Thoroughbred, Arabians’ muscles are characterized by predominance of oxidative type I fibers (25). This trait classifies Arabians as better endurance athletes due to the increased aerobic capacity and greater ability to possess energy from fatty acids. In our study, it is probable that the mechanism underlying the decreased T after the race, in yet unadapt Arabian horses, is the APR to energy deficit caused by the anaerobic pathway of metabolism that prevents efficient oxidation of fatty acids and provokes glycogen spare.

#### 4.1.2. Racehorses

Due to the profound role of T in anabolic and anti-catabolic pathways, which promote hypertrophy of the working muscle and building strength, many studies utilize this parameter as the indicator of the resistance training efficiency in humans (26–28). This hormone is found to be useful in assessing the progression of the athlete’s adaptation, because it has been observed that repeated training units affect the secretory activity of the pituitary and hypothalamus in humans (29). Increased T mediates favorable conditions for protein synthesis to meet the demand of muscle running out of building material during and shortly after exhausting, strenuous exercise. Here, studies suggest that adaptation to resistance training involves an increased response of T after exercise, which prevents immediate overreaching, that would lead to overtraining (30, 31). However, these findings are more pronounced in male athletes. In our study, racing can be considered a resistance type of training, one that utilizes mostly anaerobic pathway of energy acquisition. In consistency with current postulates, our findings indicate the role of T in racehorses’ adaptation to training. The experienced group of racehorses presented a significant increase in T level after exercise compared to no changes in the inexperienced group. This leads to the conclusion that the early recovery period after exercise is beneficial for the anabolism in experienced animals.

#### 4.1.3. Endurance horses

Since T secretion is strictly dependent on the implemented exercise mode (length and intensity), it might be true that the endurance exercise triggers a specific relation. During endurance training, the majority of energy source comes from aerobic respiration. Thus, adapting to this training involves enhanced oxygen delivery into the mitochondria and stricter regulation of muscle metabolism. Moreover, a prolonged character of endurance exercise results in more restricted energy availability throughout the activity course compared to a short bout of resistance exercise, which also influences tightened regulation by means of T secretion which influence lower energy expenditure (32).

So far, many studies report decrease in T level in endurance-trained human athletes, both men and women (33–35). There are many potential physiological bases of this effect considered, however, the most probable is the disruption of the hypothalamic–pituitary-gonadal regulatory axis. The readjustment of this axis might be the mechanism responsible for increasing the performance of endurance-trained individual where only the minimal anabolic, T-induced processes are preserved, possibly due to limited energy availability (endurance athletes have lower muscle mass) (36). Obvious discrepancies have been noted between men and women, since women show 15 times lower basal levels of T than men (30). In women, the majority of T is produced through the peripheral conversion of androstenedione and dehydroepiandrosterone (DHEA), with a smaller amount secreted from the ovaries and adrenal glands (37, 38). The anabolic effects of T are mediated through an increase in protein synthesis and a decrease in protein catabolism in the muscle fibers. This occurs through the binding of T to cytosolic receptors in target cells, leading to increased transcription of genes responsible for contractile protein synthesis (39). It has been demonstrated that endurance exercise can lead to an increase in T levels in women after prolonged endurance exercise, although the changes are not always significant (40, 41). Increases in T have been observed in both trained and untrained women, but it is important to note that differences in the exercise mode may affect the results and should not be directly compared. Additionally, studies suggest that increases in T might be related to both the intensity and duration of the exercise (42).

There is a lack of consistency in the literature on the effects of endurance exercise on blood T levels in men and women. Potential factors that may contribute to the inconsistent results observed include the timing of the T measurements (during menstrual cycle) (37, 38, 43) as well as the intensity and duration of the exercise and athlete anxiety (37). It is also worth considering that changes in the endocrine function, including T levels, can occur with age. T, like other anabolic hormones, tends to decrease over the course of life, which may impact the results of studies on the effects of endurance exercise on testosterone levels (37, 41, 44), however the differences between horses in our study were minimal. It is important to be cautious when interpreting the results of studies on this topic, as the conditions of endurance exercise can introduce many uncontrolled variables that may affect them. It is worth noting that the increase in blood T levels observed during endurance exercise may be partially attributed to a reduction in the metabolic clearance rate (MCR) of the hormone (45). MCR refers to the rate at which a hormone is metabolized and removed from the body, and it is regulated, in part, by the hepatic blood flow. During endurance exercise, hepatic blood flow may decrease, leading to a reduction in MCR and subsequently an increase in the concentration of testosterone in the blood. This phenomenon is a common characteristic of steroid hormones, including T.

In our study, endurance horses had a lower level of T after a long-distance run. There cannot be found any significant differences between the experienced and inexperienced groups. This might indicate that T is not an important factor for the endurance-training horses’ adaptation. However, the observations of the fastest equines in both groups lead to a valuable conclusion. The enhanced endurance workload in the fastest individuals resulted in the highest T-level decrease. Thus, it can be concluded that the intensity of endurance training is negatively correlated with the T level. The weakest individual expressed an insignificant drop in T-level after the competition. However, more research is needed on bigger groups of animals.

### 4.2. Cortisol

#### 4.2.1. Racehorses

C is involved in the regulation of various physiological processes such as glucose metabolism, protein synthesis, and inflammation (46). Elevated C levels can impair bone mineralization and collagen synthesis, reduce calcium absorption, and increase blood pressure (47). In addition, excessive cortisol can disrupt the balance between anabolic and catabolic hormones, leading to a reduction in T synthesis and an increased risk of exhaustion and overtraining in human athletes (46). Elevated C levels can have a range of physiological effects that help the body adapt to prolonged stimulation. However, excessive, prolonged exposure to stressors can lead to inability to trigger an adaptive response, resulting in negative consequences for the body (24). Human studies have shown that both men and women experience an increase in C levels after participating in athletic contests. It was presented that competition winners may have post-competition cortisol levels that are significantly lower than those of losers (24, 48–53). The findings of this study suggest that high-intensity, anaerobic exercise does not significantly alter C levels in racehorses, regardless of their fitness level. It is worth noting that these results may be influenced by the fact that the measurements were taken during training rather than during competition, and therefore may not accurately reflect the hormonal response to strenuous exercise during competition (24).

#### 4.2.2. Endurance horses

Similar results were observed in both groups of endurance horses, which also did not show significant changes in C concentrations in response to exercise. What should be highlighted is a significant increase observed in blood C after competition in the fastest gelding from a group of endurance, inexperienced horses. Observation made on the mares shows that the fastest individuals from both experienced and inexperienced groups displayed a significant increase in blood C after exercise, with a larger increase observed in the inexperienced group. These findings support the literature (potential losers) and prove that the response of C to exercise may differ between sexes and levels of training. What is more, our findings may suggest that less-trained horses experience hormonal imbalance and show lower capacity for adaptation to intensive training. Also, the eliminated individual had higher C concentration before and after training compared to the group average. This may suggest that pre-existing high C level may have a negative impact on performance, and that inexperienced horses may be more prone to C dysregulation in response to exercise. The analysis of changes in blood C concentrations between groups of experienced and inexperienced horses revealed that the distribution of data in the inexperienced group was more scattered and tends to show an increase in C concentrations after exercise. In contrast, among the group of experienced horses, the C concentrations were higher in the measurement taken before training. These findings highlight the potential role of C as a predictor of performance in horses, particularly considering similar studies conducted in humans. It was shown that the median C level just before training is usually higher for eventual winners than for losers, which was also confirmed in horses (24, 49–53). In addition, the higher significance of C changes in endurance horses is probably related to different energy-obtaining mechanisms involved (glycogenolysis and lipolysis which are mostly promoted by cortisol) than during race training (54).

### 4.3. T/C ratio

There is a growing body of research indicating the usefulness of the testosterone-to-cortisol (T/C) ratio as a marker of physical fitness in humans (55–58). It was postulated that the T/C ratio is more sensitive to the stress of training than either T or C measures alone. In contrast, there is a lack of similar data in equine research, where C levels are typically studied in isolation when examining performance. However, the approach of evaluating T and C levels simultaneously may be more informative in terms of assessing the effects of training on anabolic-catabolic balance in horses.

#### 4.3.1. Racehorses

Our findings suggest that experienced and inexperienced groups of racehorses present inverse patterns in the change of the anabolic index in response to training. In our research, we observed a significant decrease in the T/C ratio in the inexperienced group, and a threefold increase in the ratio in the experienced group, compared to pre-training levels. The T/C ratio is widely recognized as a marker of anabolic/catabolic balance, with high ratios indicating an anabolic state and low ratios indicating a catabolic state in human athletes (9, 10, 59). The values of the T/C ratio falling below 30% may reflect states of catabolism and overexertion, indicating greater stress and recovery time (60), while the increased is associated with a high anabolic tendency (61, 62). The T/C ratio is a well-established measure of anabolic-catabolic balance. It plays an important role in athletes, as it can influence protein synthesis and proteolysis, which are essential processes for muscle growth and repair. It is advisable for athletes to strive to increase their T/C ratio to optimize muscle protein metabolism and promote muscle growth during exercise (63). A significant decrease in the T/C index may indicate that the training intensity is too high for inexperienced horses, thus catabolic processes are prevailing, potentially leading to a decrease in skeletal muscle mass in extreme cases (55). Some research in humans has shown that there are no differences in the T/C ratio 12 h following high-intensity interval training (20 min of 15 s intervals of running at 110% of maximum oxygen consumption interspersed with 15 s of active rest at 40% of maximum oxygen consumption) (56). However, in the same study the changes were observed immediately post-intervention.

Our study (data not shown) suggests that notable differences in the response of Arabian and Thoroughbred racehorses to training exist. The T/C ratio, which is a measure of physical performance, was found to decrease in inexperienced Arabian racehorses after training. In contrast, experienced Arabian racehorses demonstrated a 2-fold increase in the T/C ratio after training. Thoroughbred racehorses, on the other hand, exhibited an increase in the T/C level after the race, among both experienced and inexperienced groups, this change was not significant. These findings suggest that there may be physiological differences between Arabians and Thoroughbreds that affect their ability to adapt to physical effort. It is possible that Arabians are less able to cope with the demands of training, while Thoroughbreds may have a greater capacity to adapt.

#### 4.3.2. Endurance horses

In endurance horses, both groups showed a decrease in the anabolic index following training, but this decrease was significant only in inexperienced horses. It should be highlighted that similar findings regarding the T/C ratio and its relationship to physical exertion have been reported in studies of human athletes participating in endurance events such as marathons, ultramarathons, and triathlons (61, 64–68). Results of our study show that in both groups, the fastest mare had a greater decline in the anabolic index compared to the fastest gelding, and all top performers exhibited a more pronounced drop in the T/C ratio below the group average, with no change in the eliminated horse. This may be correlated with the poor performance of this individual. However, it is important to consider the possibility that the timing of the blood sample collection for this individual, as the exercise ended faster, might have influenced on the result. In comparison, studies of ultramarathon athletes have demonstrated that the T/C ratio decreases immediately after the race, indicating that the body is in a catabolic state following a demanding endurance event (65–67). This decrease in the T/C ratio may be due to the stress imposed on the body by the duration and environmental conditions of the race (67). Additionally, it has been suggested that reduced serum T and T/C concentrations may be associated with fatigue and decreased maximal strength in endurance athletes (64). Another study, conducted during an Ironman Triathlon found that the T/C ratio initially increased significantly before decreasing in a significant manner until the end of the competition. The observed changes represented a 68.1% decrease in T/C ratio values between the beginning and end of the race, consistent with previous research demonstrating that intense endurance events can lead to a catabolic state in the body (68). It is important to note that the T/C ratio is often used as a marker to monitor the recovery of long-distance runners (61). However, the blood samples were obtained during standard veterinary procedure, thus other points for sampling were impossible. Numerous studies have found that the T/C ratio, a measure commonly used in training research to evaluate the athlete’s response to training stimuli, has not consistently demonstrated a connection with either training load or overtraining. This suggests that the T/C ratio may vary due to the specific nature of the sport being performed, which can lead to inconsistency in its use as an indicator of an athlete’s response to training (69, 70).

### 4.4. Serum amyloid A

#### 4.4.1. Racehorses

Serum amyloid A (SAA) is a well-established APP in the horse, known to increase in response to inflammatory stimuli or other disturbances of homeostasis (71–74). Two theories might explain the exercise-induced APR, which is the most pronounced in extreme overload and competition-related effort. Firstly, working muscles and joints undergo injuries that stimulate the pro-inflammatory cytokines, and APPs; secondly, APR might result from glycogen depletion in the muscle which promotes the pleiotropic effect of IL-6 for APPs release (16). In our study, we observed no change in SAA levels after training in experienced racehorses. However, a significant increase was noted in the inexperienced group. The fold change in SAA levels between the two groups was also significant, indicating a clear difference between the two groups. It can be hypothesized that with the horse’s adaptation to physical exertion, there is an increase in their ability to regulate the inflammatory process in the body, which may be one of the physiological mechanisms underlying the improved performance after physical conditioning observed in trained horses. In contrast, untrained horses do not exhibit the same ability to regulate inflammation, and as a result, their levels of SAA can often be higher. We were surprised by this finding, however, the increase was low (1-2-fold). Previous research has suggested that measuring SAA concentrations may be less useful for monitoring race training due to the daily nature of this type of training, which stimulates SAA production every 24 h (23). However, the number of horses in the previously mentioned study was low (*n* = 10), thus lack of statistical significance may relate to that. Moreover, studies that involved Standardbred trotters, short distance races (1600–2,500 m) also suggest no significant variation in SAA concentration after training (75). Nonetheless, another experiment of short-distance racehorses ridden at maximum speed presents a significant SAA response 24-h after exercise (76). The intensity of race training probably differed between the studies. In ours, it was the inexperienced group’s first intense gallop session, so the exertion was strenuous.

#### 4.4.2. Endurance horses

Our research demonstrated that the concentration of SAA in the blood increased following a training session in inexperienced endurance horses. This increase in SAA levels could be a result of the exercise-induced APR or an adaptive response to training (23). After endurance competition, an increase of SAA occurred but it appears that training sessions do not significantly increase SAA concentrations when the workload is appropriately promoting adaptations and maintaining health (16). It is indicated that adaptation to prolonged endurance training may be associated with a reduced APR in experienced Arabian horses (23). In our study, the fastest inexperienced individuals exhibited the greatest physiological response, as measured by SAA. The fastest mare and gelding in this group also had the highest levels of SAA following a long-distance race. This may suggest that experienced endurance horses have a better adaptation to strenuous exercise and may not exhibit APR as pronounced as the inexperienced horses. The results of the eliminated horse demonstrated a higher baseline level of SAA (before training) compared to the average of the experienced group, but it remained significantly lower than that of the inexperienced horses. It is in line with other findings suggested that SAA may be a potential indicator of the status of endurance horses (77).

Variation of SAA changes in response to different kinds of exercise in our studies might be in further support of the previous findings that postulate the correlation between high SAA concentration increase and long-duration efforts (78). It should be also taken into consideration that the type of metabolic pathway might influence the APR here, since the evidence suggests that the pro-inflammatory response has a role in acute challenge to muscle metabolism rather than its damage (79).

## 5. Limitations

Cortisol secretion is controlled by a diurnal rhythm, with levels peaking in the morning and decreasing throughout the day (80). Plasma cortisol levels reach a peak in the morning (81). In both endurance and racehorses, the blood samples of both groups were obtained at the same time for all horses. However, because endurance rides last longer, the post-exercise samples were obtained in the afternoon, max. 30 min after the exercise ended. Another limitation is the number of endurance horses, thus research on bigger groups is desired. Nonetheless, the results were similar to those obtained in human marathon runners. The blood samples were obtained immediately after the end of the race training/max. 30 min after the end of the endurance effort as a part of standard veterinary diagnostic procedures, so the longer/shorter duration of sampling was impossible. However, it is in line with sampling protocols presented in previously published studies (11, 20, 23, 24).

## 6. Conclusion

The changed levels of hormones such as T and C have been observed in horses following exercise. Considering the usability of T, C, and T/C ratio, it should be noted that changes in these parameters are different in endurance and race type activity. Moreover, these biomarkers might serve as indicators of the equine adaptation to particular type of exercise. Our study highlights that T/C ratio may serve better as a marker of performance status for racehorses rather than endurance athletes. The T/C ratio may be an unreliable indicator of overtraining in endurance exercise, that is strictly affected by the high variability of the training model where duration, volume, and intensity are highly important. As further conclusion we follow the previous recommendations that anabolic index might be more useful as an indicator of performance progress to follow up an individual over the season, regardless of the type of exercise. However, further research is needed to fully understand the mechanisms underlying this potential relationship and to confirm the utility of hormone measurement as a tool for monitoring training in horses.

## Data availability statement

The raw data supporting the conclusions of this article will be made available by the authors, without undue reservation.

## Ethics statement

Ethical review and approval was not required for the animal study because Blood samples were collected as a part of standard veterinary diagnostic procedures. Thus, no approval of the Local Commission for Ethics in Animal Experiments was required, according to the Polish legal regulations and the European directive EU/2010/6. Written informed consent was obtained from the owners for the participation of their animals in this study.

## Author contributions

OW-P: conceptualization, methodology, resources, writing—review and editing, funding acquisition, and supervision. ID, JG, KM, and OW-P: formal analysis and writing—original draft preparation. ID, JG, and OW-P: investigation. ID and JG: data curation and visualization. All authors contributed to the article and approved the submitted version.

## Funding

This research was funded partly by National Science Centre, Poland No. 2021/41/B/NZ7/03548 (OW-P).

## Conflict of interest

The authors declare that the research was conducted in the absence of any commercial or financial relationships that could be construed as a potential conflict of interest.

## Publisher’s note

All claims expressed in this article are solely those of the authors and do not necessarily represent those of their affiliated organizations, or those of the publisher, the editors and the reviewers. Any product that may be evaluated in this article, or claim that may be made by its manufacturer, is not guaranteed or endorsed by the publisher.
